# Comprehensive comparison of three different animal models for systemic inflammation

**DOI:** 10.1186/s12929-017-0370-8

**Published:** 2017-08-24

**Authors:** Semjon Seemann, Franziska Zohles, Amelie Lupp

**Affiliations:** Institute of Pharmacology and Toxicology, Jena University Hospital, Friedrich Schiller University Jena, Drackendorfer Str. 1, 07747 Jena, Germany

**Keywords:** LPS, PCI, CLP, Systemic inflammation, Oxidative stress, Cytokines

## Abstract

**Background:**

To mimic systemic inflammation in humans, different animal models have been developed. Since these models are still discussed controversially, we aimed to comparatively evaluate the most widely used models with respect to the systemic effects, the influence on organ functions and to the underlying pathophysiological processes.

**Methods:**

Systemic inflammation was induced in C57BL/6N mice with lipopolysaccharide (LPS) treatment, peritoneal contamination and infection (PCI), or cecal ligation and puncture (CLP). Blood glucose and circulating cytokine levels were evaluated at 0, 2, 4, 6, 12, 24, 48, and 72 h after induction of inflammation. Additionally, oxidative stress in various organs and liver biotransformation capacity were determined. Markers for oxidative stress, apoptosis, infiltrating immune cells, as well as cytokine expression patterns, were assessed in liver and spleen tissue by immunohistochemistry.

**Results:**

Treating mice with LPS and PCI induced a very similar course of inflammation; however, LPS treatment elicited a stronger response. In both models, serum pro-inflammatory cytokine levels rapidly increased whereas blood glucose decreased. Organs showed early signs of oxidative stress, and apoptosis was increased in splenic cells. In addition, liver biotransformation capacity was reduced and there was pronounced immune cell infiltration in both the liver and spleen. Mice exposed to either LPS or PCI recovered after 72 h. In contrast, CLP treatment induced comparatively fewer effects, but a more protracted course of inflammation.

**Conclusions:**

The LPS model of systemic inflammation revealed to be most suitable when being interested in the impact of new therapies for acute inflammation. When using the CLP model to mimic human sepsis more closely, a longer time course should be employed, as the treatment induces delayed development of systemic inflammation.

**Electronic supplementary material:**

The online version of this article (10.1186/s12929-017-0370-8) contains supplementary material, which is available to authorized users.

## Background

Sepsis remains a significant clinical challenge in intensive care units, as it frequently results in multi-organ dysfunction and high morbidity, leading to mortality rates of approximately 50% [1]. Although there are well established strategies aimed at treating the underlying infection, the development of new therapeutic options and identification of potential drug candidates are urgently needed to prevent and combat sepsis. For these purposes, animal models can be very useful. To mimic the course of human sepsis, various rodent models have been developed. These models can be classified into three major types: exogenous administration of endotoxin [lipopolysaccharide (LPS) treatment], exogenous administration of viable pathogens [inoculation with *Escherichia coli*], and disruption of the endogenous protective barrier [cecal ligation and puncture model (CLP)] [2]. All three models have advantages and disadvantages and it is still controversial as to which of these animal models is most suitable.

The LPS animal model has several essential advantages, including technical ease and high reproducibility, particularly in the inflammatory response elicited. Shortly after LPS administration, high levels of pro-inflammatory cytokines are released and can be measured in circulating serum [3]. This leads to rapid development of systemic inflammatory response syndrome (SIRS) and subsequent dose-dependent mortality [2, 4]. However, the LPS model does not exactly reproduce the characteristic features of human sepsis, with earlier and greater cytokine responses, that are shorter in duration than in humans [5, 6]. In total, the existing data suggest that LPS can be used to study the pathophysiological processes of endotoxemia or SIRS and as a model of endotoxic shock, but not of sepsis in general [5].

To compensate for the weaknesses of the LPS model, a polymicrobial sepsis model was developed. In the peritoneal contamination and infection model (PCI), stool samples from healthy, non-vegetarian donors, which contain various aerobic and anaerobic gram-positive and gram-negative bacteria, are prepared and administered intraperitoneally. Each stool sample preparation has to be microbiologically characterized, which increases the experimental effort for this model [7]. However, the advantages of this model are high reproducibility, the more or less technical ease, and induction of polymicrobial sepsis. The authors claim that the course of systemic inflammation in the PCI model is more similar to that in humans, compared to the LPS model; however, there are only a few studies published thus far on this model [4, 7]. In addition, the relevance of the PCI model to clinical sepsis is questionable, as patients rarely have massive bacteremia [5, 8] and a single application of a high dose of bacteria causes effects close to those observed after the intravenous injection of a high dose of LPS. The clinical course runs quickly – a hypodynamic circulatory state and violent rise of serum cytokine levels are observed –, and in the absence of adequate resuscitation, the outcome is early death [9].

The most widely used sepsis model is the cecal ligation and puncture (CLP) model, which is recognized to have significant compatibility to human sepsis [10, 11]. The main advantage of the CLP model is that the peritoneum is inoculated with mixed microbial flora from the animal itself through association with devitalized tissue. After CLP induction, immune, hemodynamic, and biochemical responses similar to human sepsis are produced [12]. However, the CLP model also has disadvantages. The amount of bowel leakage is difficult to control and thus, there is a wide range of variation in the sepsis outcomes. Therefore, the CLP model is not as easy to standardize as either the LPS or PCI models. Furthermore, the intestinal flora is not uniform between animals or species and comparisons between studies should be made with caution. Additionally, variations in surgical procedures and postoperative care should also be taken into account when comparing studies. For example, the position of the suture, the size of the needle, and the number of punctures can have a huge impact on the amount of pro-inflammatory cytokines released into the peritoneum and serum and on the course of the disease [13, 14].

As comprehensive comparative data on these three models are still lacking, the present study aims to thoroughly characterize the LPS, PCI, and CLP mice models over 72 h, covering the acute phase of systemic inflammation. For this purpose, a mid-grade systemic inflammation was induced which does not cause acute lethality. Thus, there was no need to perform any antibiotic treatment or fluid resuscitation of the animals, which allowed us to investigate the natural course of the disease. The systemic immune response was evaluated by measuring serum cytokine concentrations, determining parameters of oxidative stress in various organs, and tracking immune cell emigration and immigration in spleen and liver. Liver function is critical for overall patient outcome, even more than kidney and lung function [15, 16]. Therefore, liver function parameters were also determined.

All in all, this is the first study to directly and comprehensively compare these three animal models over a time period of 72 h using non-lethal methods to investigate the systemic impact and influence on organ function resulting from exposure to inflammation. As enough data already exist comparing the animal models with the clinical course in sepsis [7, 8, 10, 17, 18], we primarily did not intend to refer our results to the situation in humans. The goal of this study was to gain a better understanding of the pathophysiological processes involved in these animal models to help researchers choose the most suitable model and to determine which parameters are useful in each model when evaluating therapeutic candidates for systemic inflammation.

## Methods

### Animals and experimental procedure

The study was conducted under the license of the Thuringian Animal Protection Committee (approval number: 02–044/14). The principles of laboratory animal care and the German Law on the Protection of Animals, as well as the Directive 2010/63/EU were followed. Male adult C57BL/6N mice (12-weeks-old, body weight 25–30 g; Charles River Laboratories, Sulzfeld, Germany) were used, and the animals were housed in plastic cages under standardized conditions (light-dark cycle 12/12 h, temperature 22 ± 2 °C, humidity 50 ± 10%, pellet diet Altromin 1316, water ad libitum). Mice were treated with either lipopolysaccharides [LPS] (*Escherichia coli* 0111:B4; Sigma Aldrich, Steinheim, Germany; 5 mg/kg body weight, dissolved in 0.1 ml/10 g body weight PBS), peritoneal contamination and infection [PCI] or cecal ligation and puncture [CLP] and sacrificed as described below after 2, 4, 6, 12, 24, 48 and 72 h. At the beginning of the experiment (*t* = 0), four control mice per treatment group were sacrificed as described. These mice did not receive any treatment. The most appropriate non-lethal LPS dose was determined in several previous (pilot) studies [19]. Polymicrobial sepsis was induced by PCI and the PCI stool batch was standardized, microbiologically validated [7] and kindly donated by apl. Prof. Dr. Ralf A. Claus, Center for Sepsis Control and Care, Jena University Hospital, Jena, Germany. Mice received 1.5 μl/g body weight of the stool batch, dissolved in 0.1 ml/10 g body weight PBS, intraperitoneally, representing a non-lethal dose based on previous data [4, 7]. CLP was performed as previously described [20]. Briefly, anesthesia was induced with isoflurane and after the opening of the abdominal cavity through a midline incision along the linea alba, the cecum was identified, exposed and ligated with a nonabsorbable suture at half the distance between distal pole and the base of the cecum. Subsequently, the cecum was punctured using a 21G needle and gently compressed to extrude a small amount of cecal content. Also here, a mid-grade systemic inflammatory condition was intended. Afterwards, the abdominal cavity was closed again by a double suture. Since we were interested in the natural course of the disease and since we induced a mid-grade systemic inflammation making acute lethality very unlikely, in none of the three animal sepsis models antibiotic therapy or fluid resuscitation was given. To track the course of each animal model, investigations were performed at eight different time points. 0 h (which is equivalent to the control group), 2 h, 4 h, 6 h, 12 h, 24 h, 48 h and 72 h after inflammation onset, mice were sacrificed (each *n* = 4–6 animals per time point). At each time point, body temperatures were measured, and the condition of the animals was assessed using the Clinical Severity Score (CSS), as described previously [7]. The CSS values were assessed hourly and all efforts were made to minimize suffering of the animals. Mice which unexpectedly showed a CSS of 4 during the experimental period (which according to our experimental protocol was defined as humane endpoint) were sacrificed with an overdose of isoflurane, followed by decapitation in order to prevent further suffering. All other mice were sacrificed at the time points indicated with the same method. Brains, kidneys, livers, lungs and spleens were removed, weighed, and either fixed in 10% buffered formaldehyde or snap-frozen in liquid nitrogen for biochemical analysis. After decapitation, mice were bled completely and blood was collected in a tube (S Monovette® 1.2 ml Z Clotting activator/serum, Sarstedt, Nuembrecht, Germany) for clotting. Blood glucose levels were determined using a droplet of the whole blood with a commercially available blood glucose meter and respective test strips (BG star®, Sanofi-Aventis, Frankfurt, Germany). After 30 min, clotted blood was centrifuged at 2000 g for 10 min to obtain serum which was used for ELISA and enzymatic activity measurements. The amount of serum used for the respective assays varied between 20 μl and 50 μl. Animals that did not show any elevated levels of serum cytokines and oxidative stress in the organs were excluded from the experiment in order to make sure to have no resistant mice in the treatment groups. For histological analysis, the formalin-fixed organ samples were embedded in paraffin blocks and cut into 4-μm thin sections (*n* = 4–6 for each treatment group).

### Interleukin (IL)-6, interleukin (IL)-10, tumor necrosis factor (TNF)-α, interferon (IFN)-γ, C-X-C motif chemokine 12 (CXCL12) and alanine aminotransferase (ALAT) assay

To determine the serum levels of IL-6, IL-10, TNF-α, IFN-γ, CXCL12 and ALAT, an IL-6 Mouse ELISA Kit (Thermo Scientific, Rockford, IL, USA), an IL-10 Mouse ELISA Kit (Thermo Scientific, Rockford, IL, USA), a mouse TNF-α Quantikine ELISA kit (R&D Systems, Minneapolis, MN, USA), a mouse IFN-γ ELISA kit (Pierce Biotechnology, Rockford, IL, USA), a mouse CXCL12/SDF-1 alpha Quantikine ELISA Kit (R&D Systems, Minneapolis, MN, USA) and a EnzyChrom™ Alanine Transaminase Assay Kit (BioAssay Systems, Hayward, CA, USA), respectively, were used according to the manufacturer’s instructions.

### Oxidative status in the tissues

The tissue glutathione content in its reduced (GSH) and oxidized (GSSG) forms was analyzed by homogenizing the samples with 11 volumes of 0.2 M sodium phosphate buffer (5 mM ethylenediaminetetraacetic acid [EDTA]; pH 8.0) and four volumes of 25% metaphosphoric acid. After centrifugation (12,000 g, 4 °C, 30 min), GSH content was measured in the supernatants using a colorimetric assay, as previously described [21]. The GSSG concentration was assessed fluorometrically [22]. To determine the tissue content of lipid peroxides (LPO) as thiobarbituric acid-reactive substances (TBARS), liver samples were homogenized with 19 volumes of ice-cold saline and analyzed fluorometrically, as previously described [23]. Additionally, HO-1 (heme oxygenase 1) activities were measured in the liver 9000 g supernatants (prepared as described under ”biotransformation capacity“) using hemin as a substrate. The amount of bilirubin formed was determined photometrically and referred to the incubation time and to the protein content of the respective 9000 g supernatants [24].

### Biotransformation capacity

To obtain 9000 g supernatants for analysis, livers were homogenized with 0.1 M sodium phosphate buffer (pH 7.4) (1:2 *w*/*v*) and subsequently centrifuged at 9000 g for 20 min at 4 °C. The 9000 g supernatants were used to assess the activities of several cytochrome P450 (CYP) enzymes, and the protein content of these fractions was determined using a modified Biuret method [25]. For determination of CYP enzyme activities, the following model reactions were performed: ethoxycoumarin-O-deethylation (ECOD; [26]), ethoxyresorufin-O-deethylation (EROD; [27]), ethylmorphine-N-demethylation (EMND; [28]), methoxyresorufin-O-demethylation (MROD; [27]), and pentoxyresorufin-O-depentylation (PROD; [27]). Glutathione-S-transferase (GST) activities were determined by photometrically measuring the resulting dinitrobenzene-glutathione conjugate, GS-DNB [29].

### Histopathology and immunohistochemistry

Samples for histopathology and immunohistochemistry were prepared by cutting 4-μm sections from the paraffin blocks and floating these onto positively charged slides. Immunostaining was performed by an indirect peroxidase-labeling method, as described previously [30]. Briefly, sections were de-waxed, microwaved in 10 mM citric acid (pH 6.0) for 16 min at 600 W, and incubated with the respective primary antibodies (Table 1) at 4 °C overnight. Detection of the primary antibody was performed using either a biotinylated goat anti-rabbit, a horse anti-mouse, or a rabbit anti-goat IgG, followed by incubation with peroxidase-conjugated avidin (Vector ABC “Elite” kit, Vector, Burlingame, CA, USA). Binding of the primary antibody was visualized using 3-amino-9-ethylcarbazole (AEC) in acetate buffer (BioGenex, San Ramon, CA, USA). The sections were then rinsed, counterstained with Mayer’s hematoxylin (Sigma Aldrich, Steinheim, Germany), and mounted in Vectamount™ mounting medium (Vector Laboratories, Burlingame, CA, USA). All immunohistochemical stainings were evaluated by two independent investigators. To detect the liver glycogen content, periodic-acid-Schiff staining (PAS; periodic acid, Schiff’s reagent: Sigma Aldrich, Steinheim, Germany) was performed and to obtain an histological overview, hematoxylin and eosin staining (HE) of livers and spleens was done, using standard protocols [31, 32]. Identification of the specific cell types was based on their microscopic features along with the relative location of the cells in the respective tissues.

**Table 1:** Primary antibodies used for the immunohistochemical investigations

Primary antibody	Type, Catalogue number	Manufacturer	Dilution	Host species
CD3	monoclonal, ab16669	Abcam	1:400	Rabbit
CD8	polyclonal, sc-7188	Santa Cruz Biotechnology	1:200	Rabbit
CD68	monoclonal, ab955	Abcam	1:500	Mouse
cleaved caspase-3	monoclonal, 9661	Cell Signaling Technology	1:600	Rabbit
CXCL12	monoclonal, MAB350	R&D Systems	1:500	Mouse
CXCR4	monoclonal, 3108–1	Epitomics	1:50	Rabbit
CYP3A	polyclonal	Daiichi Pure Chemicals	1:5000	Goat
CYP2B	polyclonal	Daiichi Pure Chemicals	1:5000	Goat
CYP2E1	polyclonal	Daiichi Pure Chemicals	1:5000	Goat
F4/80	monoclonal, MCA497G	Bio-Rad Laboratories	1:200	Rat
heme oxygenase 1	polyclonal, SPA-895	Biomol GmbH	1:5000	Rabbit
iNOS	polyclonal, sc-651	Santa Cruz Biotechnology	1:500	Rabbit
TNF-α	monoclonal, sc-52746	Santa Cruz Biotechnology	1:500	Mouse

### Statistical analysis

All statistical analyses and figures were computed with GraphPad Prism software, v. 6.0 (GraphPad Software, La Jolla, CA, USA). In all cases, experiments were performed with 4–6 animals per time point of each experimental group. Statistical significance was determined by using the non-parametric Kruskal-Wallis test, followed by the Mann-Whitney U test. Statistical comparisons were made versus the control of each group and are denoted as follows: LPS (asterisk, *), PCI (plus, +), CLP (diamond, #). A *p* value <0.05 (*,+,#) was considered as statistically significant; a *p* value <0.01 (**,++,##) and a *p* value <0.001 (***,+++,###) are further specified. Data are presented as mean ± standard deviation (SD), except for the CSS values, which are presented as medians with interquartile ranges.

## Results

### Measurements of health status, including blood pressure, body temperature, and body weight, are influenced by LPS, PCI, and CLP treatment

**Fig. 1:**
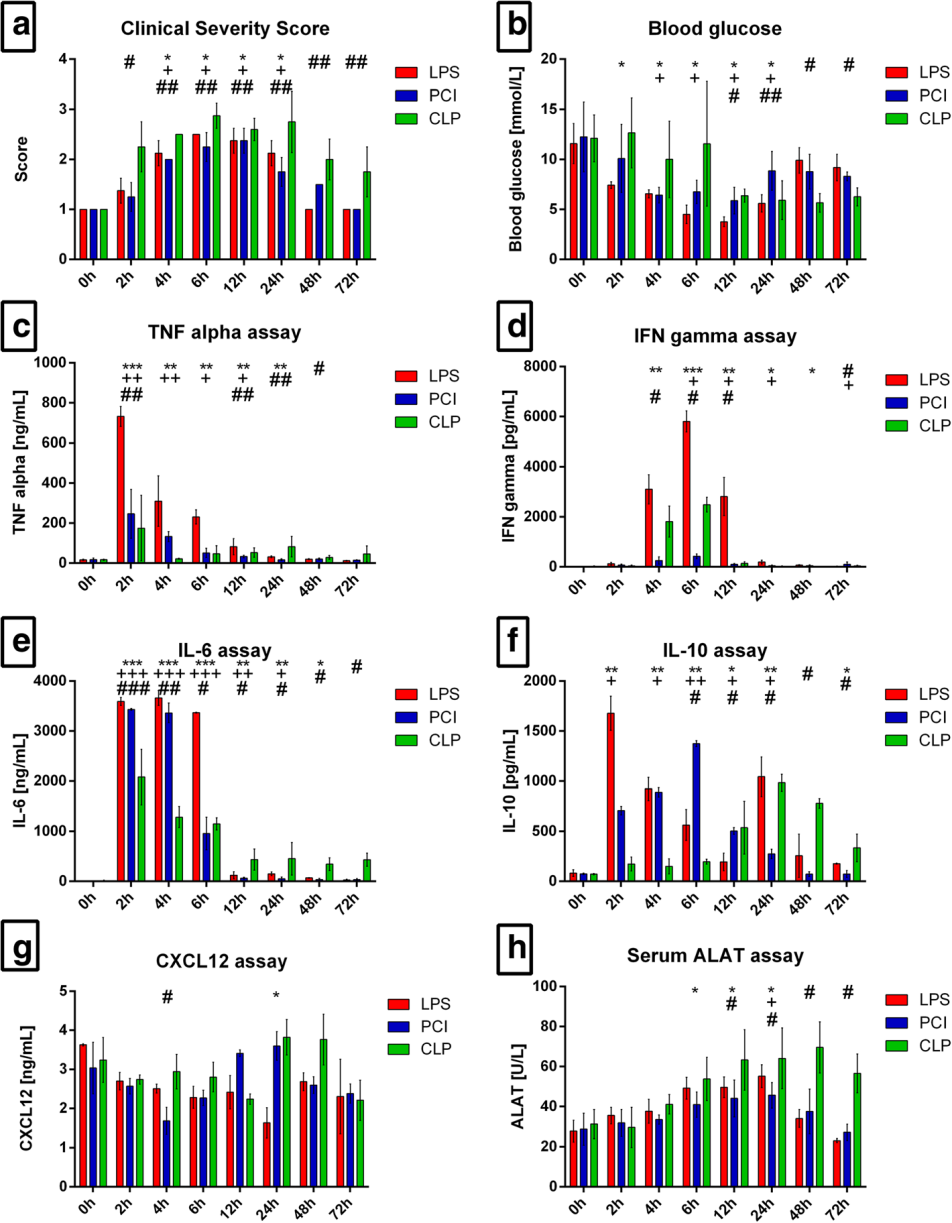
General condition and systemic parameters. Mice were treated either with LPS, PCI, CLP, or were left untreated (control). 0 h (control), 2 h, 4 h, 6 h, 12 h, 24 h, 48 h and 72 h after inflammation onset, the Clinical Severity Score (**a**) was assessed and the mice were sacrificed. Blood glucose content was determined from whole blood (**b**) and serum was obtained for TNF-α, IFN-γ, IL-6, IL-10, CXCL12 and ALAT measurements (**c-h**). Data are given as mean ± standard deviation (SD) or as median with interquartile ranges (CSS), respectively; *n* = 4–6 for each group and time point. Statistical significance was determined by using the non-parametric Kruskal-Wallis test, followed by pairwise Mann-Whitney U tests. Statistical comparisons were made versus the control of each group and are denoted as follows: LPS (asterisk, *), PCI (plus, +), CLP (diamond, #). A *p* value <0.05 (*,+,#) was considered statistically significant; a *p* value <0.01 (**,++,##) and a *p* value <0.001 (***,+++,###) are further specified

In each animal model we induced a mid-grade, non-lethal, systemic inflammatory condition to investigate the course of the disease. In the CLP model, however, one mouse after 24 h, one after 40 h and one after 70 h, had to be euthanized because of having unexpectedly reached a CSS of 4. Because these animals did not match to the allotted time points, they were not included in the further analyses. Mice receiving LPS or PCI displayed impaired health status, as evidenced by increased clinical severity scores (CSS) compared to controls (Fig. 1a). The maximum values were reached by 6–12 h after induction of infection, followed by a decline to baseline levels. After 48–72 h, there were no detectable differences in CSS values between treated mice and respective control animals. Mice subjected to CLP also exhibited peak CSS values after 6–12 h, but in contrast to the other two groups of animals the values remained elevated up to 72 h, indicating a protracted course of the disease without recovery (Fig. 1a).

Blood pressure and heart rate changes are of vital importance during systemic inflammation. Therefore, these parameters were measured at 24 h in order to get an impression of how these parameters are affected by the different treatment modalities. In all three animal models, blood pressure values decreased (Control: 115 ± 6 mmHg, LPS: 80 ± 8 mmHg, PCI: 90 ± 16 mmHg, CLP: 79 ± 16 mmHg) whereas the average heart rate increased (Control: 446 ± 51 bpm, LPS: 591 ± 33 bpm, PCI: 514 ± 60 bpm, CLP: 554 ± 43 bpm), in comparison to the control groups. However, there were no significant differences between the three animal models (Additional File 1).

Body temperature and weight are also affected during systemic inflammation. LPS, PCI, and CLP treatment induced a reduction in body temperature as well as body weight in comparison to control mice. Body temperatures largely decreased between 2 and 12 h, with the greatest decrease in the CLP-treated mice (Additional File 1). After 6 h, hypothermia was observed in these animals (CLP: 30.20 ± 4.05 °C vs. control: 37.54 ± 0.50 °C, *p* = 0.020). The effect of the different treatment modalities on body weight was very similar, as the mice in all three animal models lost weight up to 24 h (vs. control: LPS −2.60 ± 0.78 g, *p* = 0.03; PCI -1.90 ± 1.48 g, *p* = 0.20; CLP -2.05 ± 0.90 g, *p* = 0.02). After 24 h, the PCI- and CLP-treated mice regained weight, whereas the LPS-treated group had still reduced weights in comparison to controls at 48 and 72 h post-treatment (Additional File 1).

### Blood glucose, serum cytokine, and liver enzyme levels vary with sepsis model

To further determine the systemic consequences of LPS, PCI, and CLP treatment, we assessed glucose levels in whole blood, as well as inflammatory cytokines levels in serum. As shown in Fig. 1b, LPS- and PCI-treated mice exhibited hypoglycemia, which reached its peak 12 h post-infection. After 12 h, blood glucose levels continuously rose, eventually recovering almost to control levels. In the CLP model, hypoglycemia was also seen, but in contrast to the LPS and PCI models, blood sugar levels remained lower up to 72 h post-infection (Fig. 1b).

Inflammatory cytokine serum concentrations varied between the three animal models. LPS administration induced an elevation in tumor necrosis factor (TNF)-α levels by approximately 4500% compared to controls after only 2 h (Fig. 1c). In contrast, PCI and CLP treatment raised TNF-α concentrations only by approximately 1500% and 1000%, respectively, when compared to the controls. The further the course of the disease, the less TNF-α was measured. However, the CLP group exhibited elevated TNF-α serum levels at later time points (Fig. 1c). Similar as with TNF-α, LPS treatment had the strongest effect on serum interferon (IFN)-γ levels compared to the other models (Fig. 1d). Markedly increased levels were measured after 4, 6, and 12 h (to approximately 25,000%, 45,000%, and 22,000%, respectively) compared to control values. Whereas CLP treatment resulted in moderate increases in IFN-γ, PCI administration showed almost no effect on serum IFN-γ concentrations (Fig. 1d). Examining interleukin (IL)-6 concentrations, endotoxin administration led to an early increase in serum IL-6 levels especially at 2, 4, and 6 h post-infection (Fig. 1e). PCI-treated mice showed a similar increase in the serum levels. CLP treatment also resulted in an early elevation of the cytokine, but the increase was less than observed in the LPS or PCI model. However, after CLP treatment elevated IL-6 levels were still detectable later in the course of infection, at 72 h, when IL-6 concentrations were still approximately 5000% higher in comparison to control mice (Fig. 1e).

To investigate compensatory mechanisms, serum IL-10 concentrations were also measured. Here, the three animal models elicited varying results. LPS treatment induced a biphasic IL-10 response, with a large increase after 2 h, followed by a decrease up to 12 h, and a second elevation after 24 h (Fig. 1f). PCI treatment led to a continuous increase in IL-10 levels up to 6 h, followed by a step-by-step reduction, whereas CLP-treated mice showed a monophasic course with higher IL-10 levels at later time points.

As the CXCR4/CXCL12 axis has been shown to be of diagnostic as well as of overall importance in inflammation [19, 33, 34], the amount of serum CXCL12 was assessed. In the LPS-treated mice, CXCL12 levels continuously decreased up to 24 h after inflammation onset, followed by an increase at later time points. PCI treatment, in contrast, induced a biphasic response, with lower levels of CXCL12 after 4 h, and increased levels after 24 h, compared to control. CLP treatment led to elevated serum CXCL12 levels after 24 and 48 h (Fig. 1g).

To determine to what extent liver integrity was influenced by the three treatment modalities, serum alanine transaminase (ALAT) levels were measured. Whereas the LPS and PCI groups exhibited the highest ALAT concentrations after 24 h, CLP treatment led to a continuous increase in ALAT levels past 24 h (Fig. 1h).

### LPS, PCI and CLP treatment influence tissue oxidative stress markers differentially

**Fig. 2:**
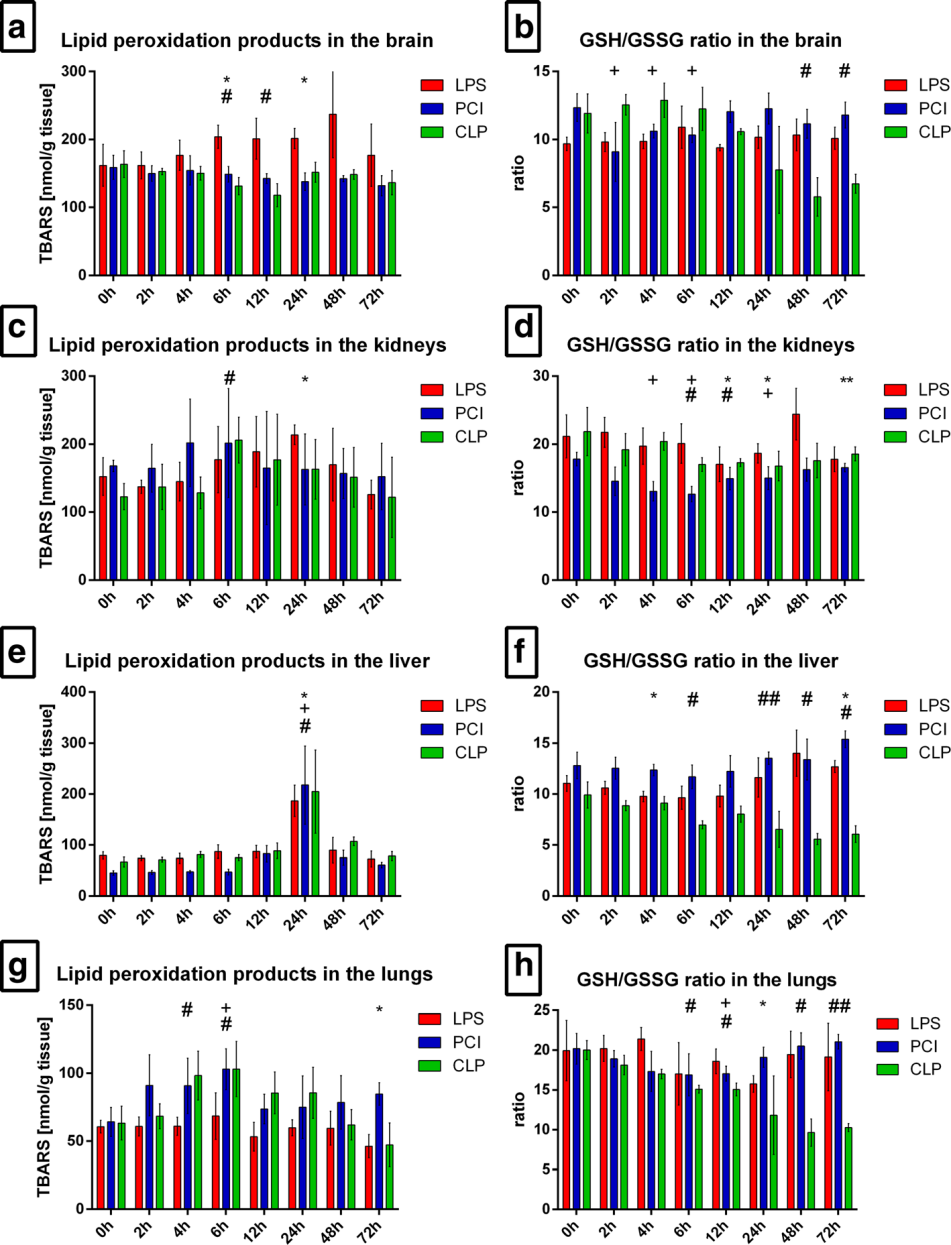
Oxidative stress in different organs. At the time point indicated, mice were sacrificed and different organs were collected for the analysis of the tissue content of lipid peroxidation products as determined by thiobarbituric acid reactive substances (TBARS) (**a**, **c**, **e, g**). Additionally, the glutathione status was assessed and the GSH/GSSG ratio was calculated (**b**, **d, f, h**). Data are given as mean ± standard deviation (SD), *n* = 4–6 for each group and time point. Statistical significance was determined by using the non-parametric Kruskal-Wallis test, followed by pairwise Mann-Whitney U tests. Statistical comparisons were made versus the control of each group and are denoted as follows: LPS (asterisk, *), PCI (plus, +), CLP (diamond, #). A *p* value <0.05 (*,+,#) was considered statistically significant; a *p* value <0.01 (**,++,##) and a *p* value <0.001 (***,+++,###) are further specified

As oxidative stress has a substantial impact on organ function and may serve as a marker for inflammation severity, we next assessed the oxidative status of various organs through quantification of lipid peroxidation products (LPO), as well as both reduced glutathione (GSH) and oxidized glutathione (GSSG) in the brains, kidneys, livers, and lungs of treated and control mice. LPS treatment induced LPO production in brain tissue, starting at 4 h post-exposure. Neither PCI nor CLP treatment affected LPO values to a similar extent (Fig. 2a). However, LPS treatment did not influence glutathione status in the brain, as GSH and GSSG remained at constant levels throughout the course of inflammation. Likewise, PCI did not affect glutathione status. However, the CLP procedure led to decreased GSH/GSSG ratios after 48 and 72 h (Fig. 2b).

In the kidneys, LPS-treated mice exhibited the highest LPO concentrations after 24 h, whereas both PCI and CLP treatment induced maximum values at 6 h post-infection (Fig. 2c). All three treatments led to decreased GSH/GSSH ratios in the kidneys, with significantly reduced ratios at 4–24 h post-infection (Fig. 2d). Total glutathione content increased at later time points after LPS and PCI treatment, whereas CLP-treated mice showed no differences to the controls (Additional File 2).

In the livers, no difference in LPO response was detectable between the three model groups. At 24 h, LPO levels were increased and then returned to baseline values (Fig. 2e). Glutathione status, in contrast, was diversely affected. Whereas LPS and PCI treatment induced a decrease in the ratio 4–6 h post-infection and an elevation at later time points, CLP treatment led to a step-by-step decrease in the ratio, with minimum values after 48 h (Fig. 2f). All three treatments resulted in lowered total liver glutathione content after 6–24 h. In concordance with other results, total liver glutathione in LPS- and PCI-treated mice increased at later time points, whereas the CLP procedure resulted in reduced levels even after 72 h (Additional File 2).

In the lungs, LPS administration had no effect on LPO values, whereas PCI and CLP treatment induced an increase, with maximal LPO levels observed at 6 h post-infection. The GSH/GSSG ratio in the lungs of the LPS and PCI groups was distinctly lower after 24 h or 12 h, respectively, when compared to controls. Also here, at later time points control values were reached again in the LPS and PCI models, while CLP treatment resulted in a continuous decrease in the lung GSH/GSSG ratio, reaching minimum values at 48 h (Fig. 2g and h).

Since every single mouse exhibited elevated levels of inflammatory cytokines and oxidative stress in various organs, we conclude that no mouse has been resistant to LPS, PCI and CLP treatment, respectively.

### Liver function is affected in the three models of systemic inflammation

**Fig. 3:**
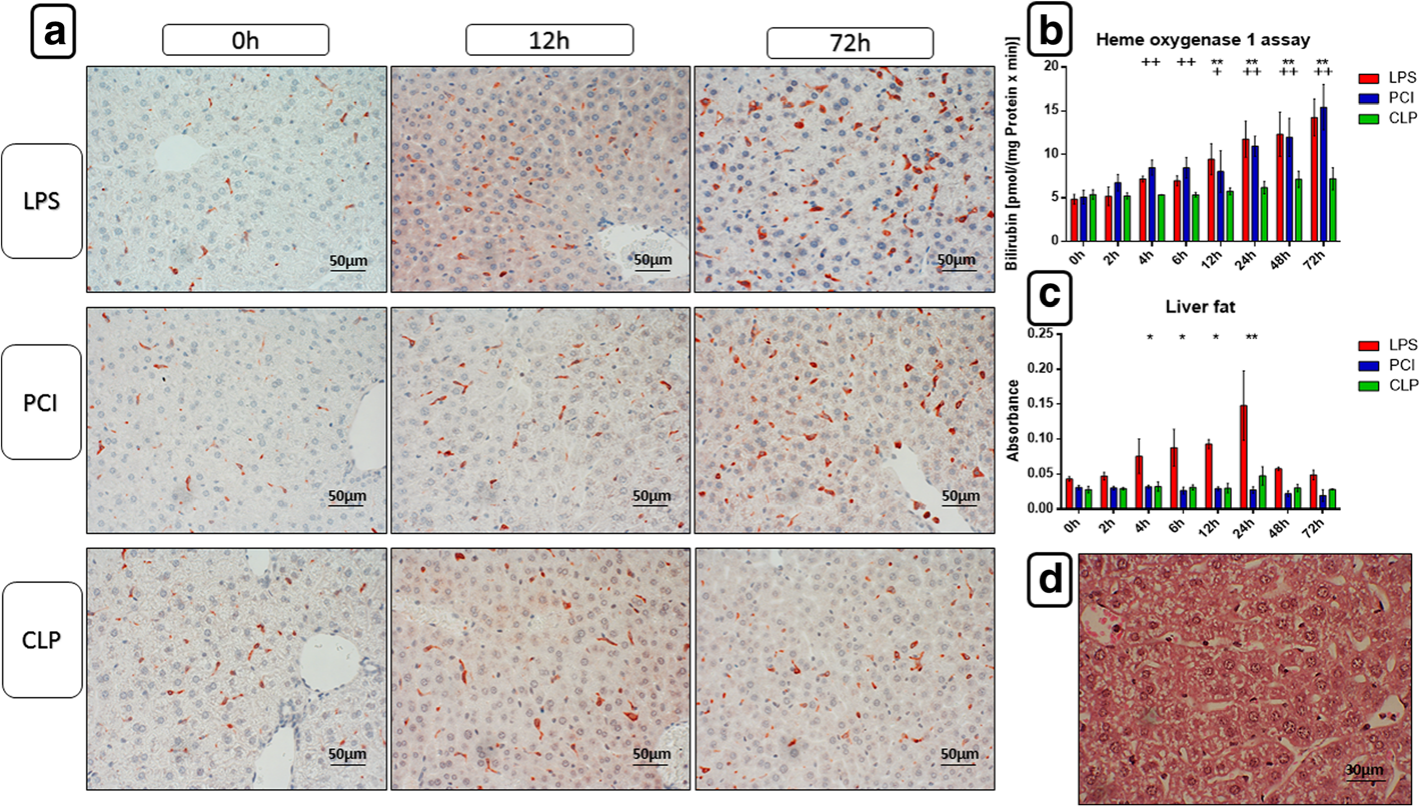
Heme oxygenase-1 expression and activitiy and total fat content in the livers. At the time point indicated, mice were sacrificed and livers were collected for further biochemical and histological analysis. Representative photomicrographs from one of 4–6 different tissue samples stained for HO-1 expression at 0 h, 24 h and 72 h after LPS, PCI or CLP treatment are shown (immunohistochemistry (red-brown color), counterstaining with hematoxylin, original magnification: (**a**, 400×)). Additionally, HO-1 activities were assessed in the liver 9000 g supernatants of liver homogenates as described in the Materials and Methods section (**b**). As an approximate measurement for the fat content, the turbidity value in the 9000 g supernatants of the livers was determined (**c**) and HE stainings were performed. The photomicrograph in (**d**) shows a representative liver of an endotoxin treated mouse after 24 h, displaying large amounts of fat droplets. Data are given as mean ± standard deviation (SD), *n* = 4–6 for each group and time point. Statistical significance was determined by using the non-parametric Kruskal-Wallis test, followed by pairwise Mann-Whitney U tests. Statistical comparisons were made versus the control of each group and are denoted as follows: LPS (asterisk, *), PCI (plus, +), CLP (diamond, #). A *p* value <0.05 (*,+,#) was considered statistically significant; a *p* value <0.01 (**,++,##) and a *p* value <0.001 (***,+++,###) are further specified

Because ALAT levels were increased in the serum of all three animal groups, liver function was further examined to gain a better understanding of the underlying processes. Hematoxylin and eosin (H&E) as well as periodic acid-Schiff (PAS) staining were performed to analyze liver tissue sections. H&E staining revealed a massive fat accumulation as well as infiltration of inflammatory cells in the livers of LPS-treated mice after 24 h (Fig. 3c and d). Although PCI- and CLP-treated mice did not show liver fat accumulation (Additional File 2), infiltrating cells were also found. However, the infiltration was less pronounced in comparison to endotoxin treatment.

When determining the liver protein content as a reference for the cytochrome P450 (CYP) model reactions, the turbidity value of each sample was assessed as an approximation of the liver fat content. In agreement with the histological data, turbidity values were increased after LPS treatment at 4–24 h only (Fig. 3c and Additional File 2).

As a measure of the liver glycogen content, PAS staining revealed a similar response in all three animal models. Treatment with LPS, PCI, and CLP led to a distinct loss of glycogen in the livers after 6 h. This effect was still present at 24 h, but levels recovered after 24 h to reach control values by 72 h in all models.

As an additional measure of oxidative stress in the livers, heme oxygenase-1 (HO-1) and inducible nitric oxide synthase (iNOS) expression were determined by immunohistochemistry. Shown in Fig. 3a, Kupffer and pit cells expressed HO-1 to a similar extent in LPS and PCI treatment groups. As the time post-treatment progressed, HO-1 expression increased, with intense expression seen after 72 h. In contrast, after CLP treatment, HO-1 expression was not affected. To substantiate these results, HO-1 activity was measured in liver 9000 g supernatants. As shown in Fig. 3b, as with HO-1 expression, there was an increase in enzyme activity progressing after LPS or PCI treatment, whereas CLP had no impact on HO-1 activity.

**Fig. 4:**
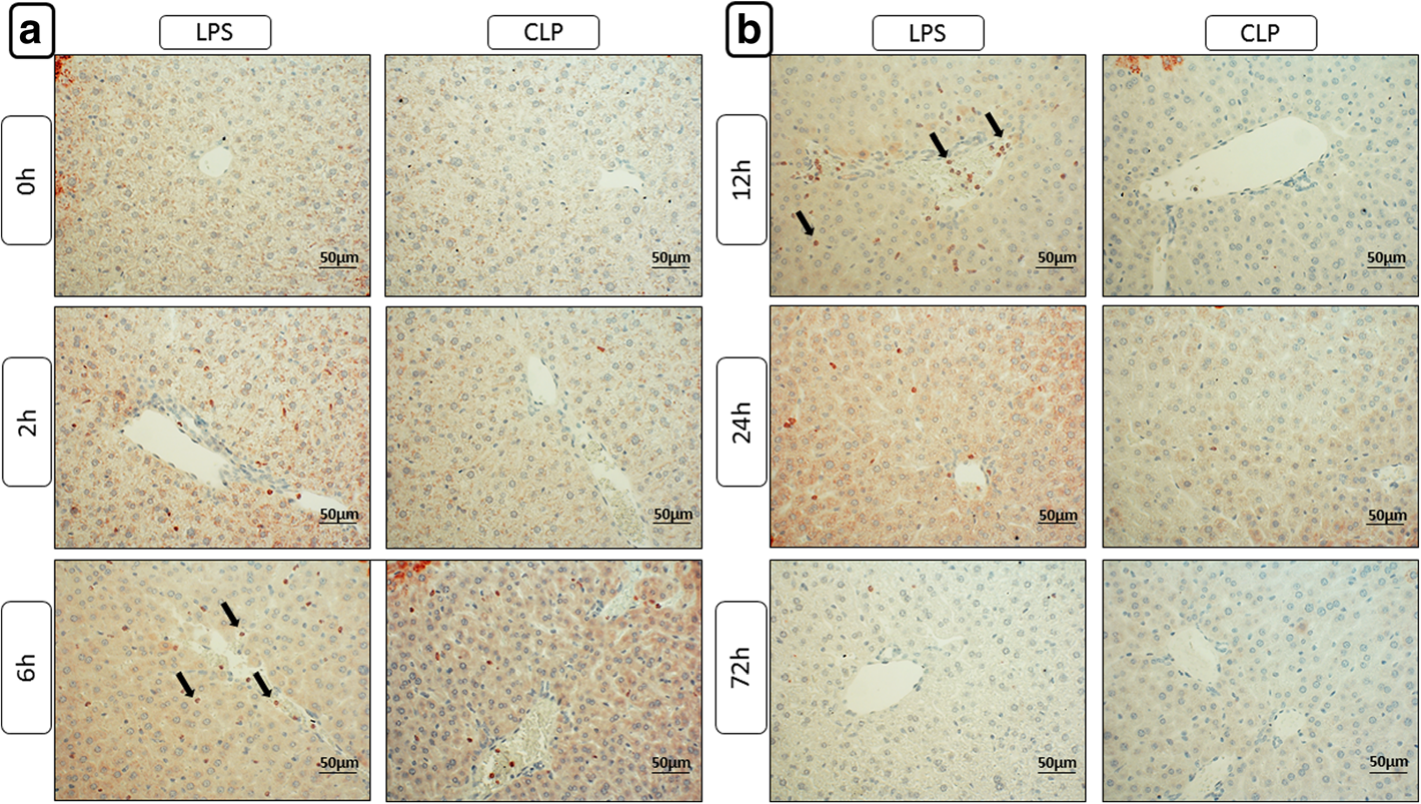
Immunohistochemical evaluation of iNOS expression. At the time point indicated, mice were sacrificed and the livers were collected for immunohistochemical analysis of iNOS expression (red-brown color, counterstaining with hematoxylin). Representative photomicrographs from one of 4–6 different tissue samples each are shown (original magnification: (**a**, **b**) 400×, arrows are used to exemplarily show iNOS expressing infiltrating neutrophil granulocytes). The time points 0 h, 4 h, 12 h, 24 h and 72 h were chosen as representative time points to show the course. The staining results after PCI treatment are not depicted separately but can be found in the additional file 3 as the course was very similar to that observed after LPS administration, with the only difference, however, that less iNOS positive cells are to be seen

Upon examining iNOS expression, there were no major differences between the LPS and PCI groups; however, the PCI treatment effect was less pronounced than that of LPS (Fig. 4 and Additional File 3). Administration of endotoxin led to an infiltration of iNOS-positive neutrophil granulocytes into the periportal regions of the liver lobules starting at 2 h post-infection. The largest number of infiltrating granulocytes was observed after 12 h and spread throughout the liver lobules. At later time points, few granulocytes were detectable. This effect on neutrophil migration following LPS and PCI treatment exceeded that seen after CLP treatment, where at 6 h only several neutrophils were observed, without further increases as the disease progressed (Fig. 4a, b and Additional File 3).

To gain more insight into the nature of the resident and infiltrating immune cells in the livers, sections were stained for the cell markers, CD68, F4/80, and CD3. CD68-positive cells were scarcely observed in the livers of all three treatment groups, without any significant difference to the controls. In contrast, strong F4/80 expression was seen in Kupffer and pit cells. Especially LPS and PCI challenge led to an intense increase in F4/80 expressing cells after 48 h and 72 h, an effect, which was not as pronounced after CLP treatment (Additional File 2). No observable differences were detected also in CD3 expression between the three treatment groups. Immediately after inflammation initiation, all groups had an increase in CD3+ cells throughout the liver lobules, with the maximum effect seen after 48–72 h. In the periportal regions of the liver lobules, the largest number of CD3+ lymphocytes was detectable. Here, the maximum effect was observed after 72 h.

**Fig. 5:**
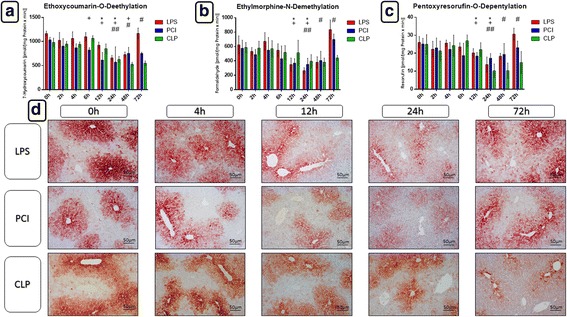
Biotransformation capacity in the livers. At the time point indicated, mice were sacrificed and the livers were collected for biochemical and histological analysis. Ethoxycoumarin-O-deethylation [ECOD] (**a**), ethylmorphine-N-demethylation [EMND] (**b**) and pentoxyresorufin-O-depentylation [PROD] (**c**) activities in 9000 g supernatants are shown exemplarily. Data are given as mean ± standard deviation (SD), *n* = 4–6 for each group and time point. Statistical significance was determined by using the non-parametric Kruskal-Wallis test, followed by pairwise Mann-Whitney U tests. Statistical comparisons were made versus the control of each group and are denoted as follows: LPS (asterisk, *), PCI (plus, +), CLP (diamond, #). A *p* value <0.05 (*,+,#) was considered statistically significant; a *p* value <0.01 (**,++,##) and a *p* value <0.001 (***,+++,###) are further specified. **d**: CYP 3A2 isoforms expressions in liver tissue as determined by immunohistochemistry (red-brown color, counterstaining with hematoxylin). Representative photomicrographs from one of 4–6 different liver tissue samples are shown (original magnification: 400×). 0 h, 4 h, 12 h, 24 h and 72 h were chosen as representative time points to depict the course

One central parameter of liver function is its biotransformation capacity. LPS, PCI, and CLP treatment induced a significant and continuous loss in CYP enzyme activity in the liver. Twenty-four hours after infection onset, both endotoxin and PCI models had decreased activity of the CYP families 1A, 2A, 2B, and 2C (ECOD) to approximately 55% of control values, whereas CLP treatment reduced the activities to approximately 65% of controls (Fig. 5a). Similar results were measured with the individual activities of CYP1A (EROD), CYP1A2 (MROD), CYP3A (EMND), and CYP2B (PROD) (Fig. 5b and c). Notably, when treating mice with LPS and PCI, CYP activities returned to control levels after 72 h, whereas after CLP treatment, activities remained consistently reduced at later time points. These results were further confirmed by immunohistochemistry. As shown in Fig. 5d, CYP enzymes were predominantly expressed around the central veins of the liver lobules. At 12–24 h after LPS or PCI treatment, CYP expression was remarkably decreased, but returned to normal levels after 72 h. In contrast, enzyme expression remained diminished for up to 72 h with CLP treatment.

As a representative for phase 2 enzyme activities, glutathione-S-transferase (GST) activity was measured. No differences were detectable between the three treatment groups. In all animal models GST activity continuously declined during the course of the disease and remained significantly diminished at 72 h (Additional File 2).

### Splenic morphology and histology is influenced differentially in the sepsis models

**Fig. 6:**
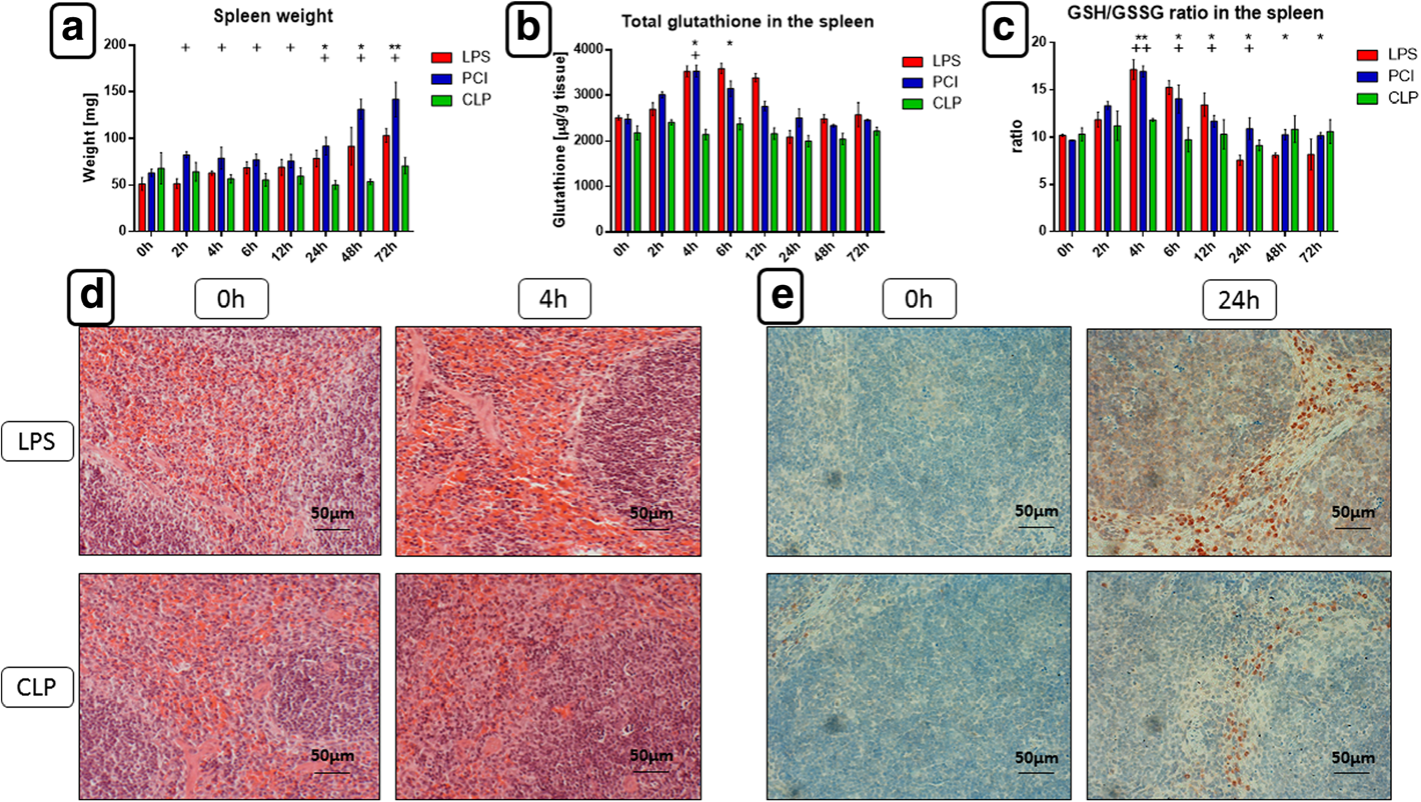
Spleen weights, glutathione status, histomorphology and iNOS expression in the spleen. At the time point indicated, mice were sacrificed and the spleens were collected for further analysis. (**a**) Spleen weights. (**b, c**) Total glutathione content and GSH/GSSG ratio in the spleen tissue. Data are given as mean ± standard deviation (SD), *n* = 4–6 for each group and time point. Statistical significance was determined by using the non-parametric Kruskal-Wallis test, followed by pairwise Mann-Whitney U tests. Statistical comparisons were made versus the control of each group and are denoted as follows: LPS (asterisk, *), PCI (plus, +), CLP (diamond, #). A *p* value <0.05 (*,+,#) was considered statistically significant; a *p* value <0.01 (**,++,##) and a *p* value <0.001 (***,+++,###) are further specified. (**d**) HE staining showing a large amount of erythrocytes in the red pulp of endotoxic mice after 4 h and a less pronounced effect after CLP. (**e**) iNOS expression in the spleens, 24 h after LPS and CLP treatment (immunohistochemistry [red-brown color], counterstaining with hematoxylin). Representative photomicrographs from one of 4–6 different tissue samples each are shown (original magnification: 400×)

The spleen functions to clear senescent erythrocytes and maintain a blood reserve, and plays a significant role in the immune system. Therefore, we next investigated its response to the different inflammatory challenge models. LPS and PCI treatments caused a time-dependent splenomegaly. In comparison to controls, LPS and PCI challenge significantly increased splenic weights by approximately 100% and 125%, respectively, after 72 h. CLP treatment, however, did not affect splenic weight (Fig. 6a). Histological examination revealed an increased number of erythrocytes in the red pulp after 4 h, along with mild edema in the LPS and PCI models, whereas the impact of CLP was not that evident (Fig. 6d and Additional File 3). Additionally, iNOS positive neutrophils were detected in the red pulp at 24 h post-LPS administration and post-PCI (Fig. 6e and Additional File 3). After 24 and after 48 h, neutrophils were also detectable in the white pulp of endotoxic mice. However, this infiltration was not observed in the other two animal models.

Splenic oxidative status was also determined by measuring the content of reduced and oxidized glutathione. GSH/GSSG ratios, as well as total glutathione, increased 4 h after LPS or PCI challenge (Fig. 6b and c). At later time points, however, there were no differences observed in total glutathione content, whereas the GSH/GSSG ratio in endotoxin-treated mice was decreased in comparison to controls, indicating higher oxidative stress (Fig. 6b and c).

**Fig. 7:**
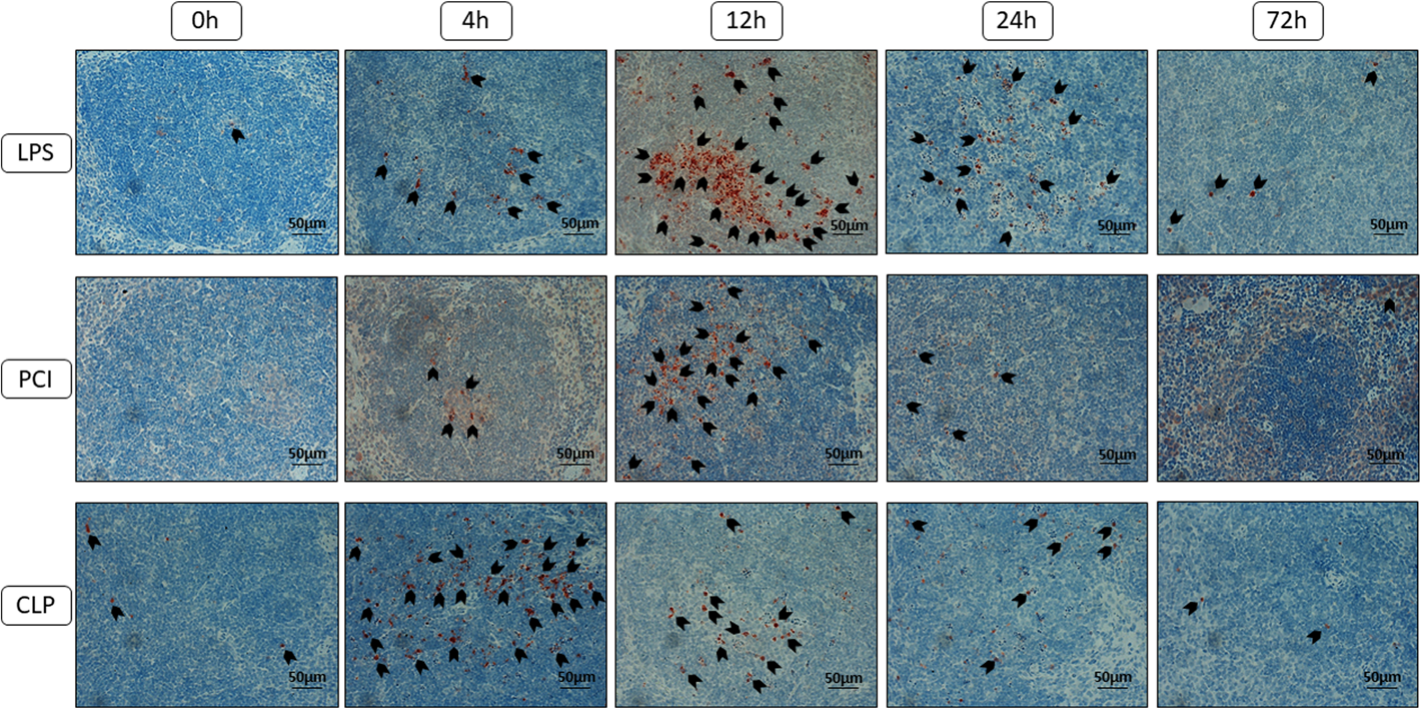
Cleaved caspase-3 expression in the spleens. At the time point indicated, mice were sacrificed and the spleens were collected for immunohistological analysis. Representative photomicrographs from one of 4–6 different tissue samples stained for cleaved caspase-3 expression are shown (red-brown color, counterstaining with hematoxylin; original magnification: 400×). 0 h, 4 h, 12 h, 24 h and 72 h were chosen as representative time points to depict the course. Furthermore, arrowheads were added to obtain an overview about the amount of cells expressing the enzyme

Apoptosis plays an important role in inflammation. Therefore, we determined the amount and distribution of apoptotic cells by immunohistochemical analysis of the cleaved form of the pro-apoptotic enzyme, caspase-3. As depicted in Fig. 7, the majority of cells expressing cleaved caspase-3 was located in the white pulp. LPS challenge upregulated this expression at 12 and 24 h noticeably, in comparison to controls and the other treatment groups. CLP treatment induced the appearance of tingible body macrophages as soon as 2 h post-treatment, with cleaved caspase-3 expression peaking already after 4–6 h, indicating early apoptotic events.

**Fig. 8:**
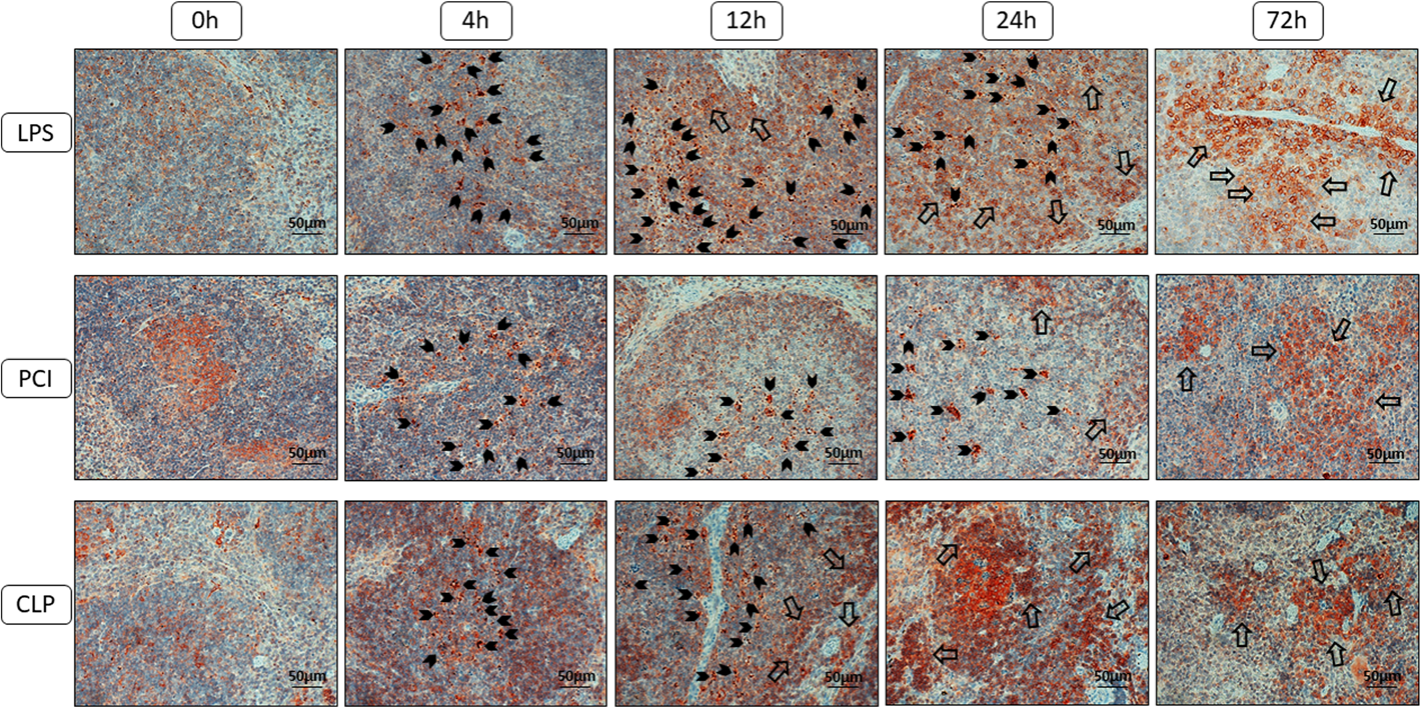
CXCR4 expression in the spleens. At the time point indicated, mice were sacrificed and the spleens were collected for immunohistological analysis. Representative photomicrographs from one of 4–6 different tissue samples stained for CXCR4 expression are shown (red-brown color, counterstaining with hematoxylin; original magnification: 400×). 0 h, 4 h, 12 h, 24 h and 72 h were chosen as representative time points to depict the course. Additionally, arrowheads were added to mark the CXCR4 positive cells that were engulfed by the tingible body macrophages and larger arrows were used to show the infiltrating immune cells exhibiting membrane-bound CXCR4 expression at later time points

To compare our previous findings [33] and the serum CXCL12 concentrations of the present investigation with the splenic chemokine expression, splenic sections were stained for CXCR4 and CXCL12. In control animals, CXCR4 was expressed on macrophages and lymphocytes in the red and white pulp, and on lymphocytes in the marginal zone of lymphoid follicles. Early after LPS treatment, CXCR4+ cells were present in the follicles inside tingible body macrophages. Similar observations were made after PCI and CLP treatment, with maximal staining reached at 12–24 h post-infection. After 72 h, endotoxin and PCI treated mice exhibited a large number of CXCR4+ cells in the red pulp, particularly around the vessels, with a similar but less degree of staining seen with CLP treatment (Fig. 8).

**Fig. 9:**
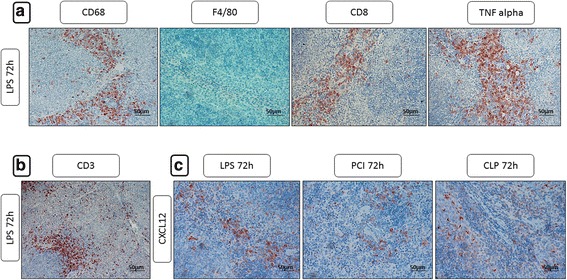
CD68, F4/80, CD8, TNF-α, CD3 and CXCL12 expression in the spleens after 72 h. At the time point indicated, mice were sacrificed and the spleens were collected for immunohistological analysis. Representative photomicrographs from one of 4–6 different tissue samples stained 72 h after inflammation onset for CD68, F4/80, CD8, TNF-α, CD3 and CXCL12 expression are shown (red-brown color, counterstaining with hematoxylin; original magnification: 400×)

To examine the cell populations having immigrated into the red pulp of the spleens after 72 h in greater detail (remarkable immune cell immigration was only visible after 48–72 h), additional immunohistochemical stainings were performed. Here, the differences between the three treatment groups were secondary to us. However, by using CXCL12 as a marker for immune cells, we observed a significant amount of CXCL12-expressing monocytes in the red pulp 72 h after LPS challenge. PCI and CLP treatment, in contrast, did not induce CXCL12 expression to a similar extent (Fig. 9c). As stated previously, iNOS+ cells were present in the red pulp after 24 h of LPS treatment. Although weaker staining was observed, iNOS+ neutrophils remained after 72 h. Additionally, a distinct set of CD68+, but F4/80-negative cells were found in the red pulp. As depicted in Fig. 9a, CD68+ cells were also observed in the white pulp of the spleens of LPS-treated mice, but not in the other models. CD8+ cells were observed in both the red and the white pulp 72 h after LPS administration. In spleens of PCI and CLP-treated mice, the red pulp also had CD8+ cells, although to a lesser extent than in LPS-treated animals. Notably, cells in the red pulp were not predominantly CD3+ (Fig. 9b). Finally, TNF-α was strongly expressed in the cell population which had immigrated into the red pulp after 72 h, with more pronounced staining after LPS administration as compared to either PCI or CLP treatment.

## Discussion

The objective of this study was to comparatively assess the time courses of different pathophysiological parameters in three commonly used animal sepsis models, LPS, PCI, and CLP. The models showed both similarities as well as distinct differences, with the most common notable differences observed between the CLP treatment group and the LPS/PCI models. LPS- and PCI-treated animals had similar courses of systemic inflammation and measurable outcomes. Since the levels of pro-inflammatory cytokines and oxidative stress were elevated in every single mouse, we conclude that the systemic inflammation was successfully induced and that mice were not resistant to LPS, PCI or CLP treatment.

Although a non-lethal systemic inflammation model was intended, following CLP treatment three mice had to be sacrificed before the end of the experiment due to having reached a CSS of 4, demonstrating a disadvantage of this experimental model. Standardization is known to be difficult with CLP methodology and is dependent on the technique used and the experience of the surgeon. Indeed, in the current study, the CLP group had the largest standard deviations in most outcome measurements. In addition, mice that had received CLP treatment exhibited impaired health status at the later time points investigated, indicating a protracted course of systemic inflammation. These results are in agreement with several other studies, suggesting that CLP treatment induces a delayed course of disease [3, 35]. In contrast, LPS and PCI treatment induced an acute inflammatory condition that progressed quickly, but also resolved rapidly, as after 72 h, mice returned to a healthy status. Since negative outcomes after LPS and PCI treatment have been reported to occur mainly during the first 72 h [36, 37], it is very likely that all mice will survive in the long-term. In contrast, on the basis of our findings and on literature data, we expect only few mice to further survive CLP treatment post-72 h [20]. As a result of an acute and severe systemic inflammation, mice in all models consumed less food and water, leading to a significant, but transient weight loss, albeit to a lesser extent in the CLP group, confirming previous results [38]. Additionally, LPS and CLP administration resulted in hypothermia, as in other studies [38]. This was probably a result of the rapid induction of the disease state and the large surface-area-to-volume ratio of these small animals [18].

In addition to general health status, there were significant differences in the kinetics and magnitude of the serum cytokine release between the three animal models. LPS and PCI treatment induced early peak cytokine responses of TNF-α and IL-6 with a rapid decrease in values thereafter. In contrast, a previously published study reported that PCI treatment caused only a negligible TNF-α response and a delayed release profile for IL-6, increasing over several hours [4]. This apparent discrepancy could be explained by the higher PCI dose used in the literature study (thus making fluid resuscitation necessary), as the authors aimed to induce mortality after 48 h. The PCI model is a relatively new model and, consequently, inter-laboratory differences have to be considered. With respect to human sepsis, IL-6 levels have been shown to reflect the severity of disease and to correlate with mortality [39], and the persistence of IL-6 and TNF-α rather than the peak levels have been shown to correlate with disease severity [40], suggesting that the CLP model is most comparable to human sepsis with respect to cytokine response.

The exogenous administration of LPS is associated with a massive toll-like receptor 4 (TLR4) activation, leading to a significant cytokine response. Presumably, the application of a polymicrobial suspension does not trigger TLR4 activation directly to the extent seen with LPS, but our results also indicate that the bacteria and their components evoked rapid onset of inflammation. Accordingly, in our investigation the differences seen between the LPS and PCI group with respect to the course of inflammatory cytokine release were not very conspicuous. CLP treatment, in contrast, is associated with a comparably slow release of bacteria into the bloodstream, thus showing a slower but protracted course of the disease.

All animal models resulted in reduced blood glucose levels and low liver glycogen reserves early after inflammation onset, which is in accordance with previous investigations [41, 42]. However, the glucose kinetics of all animal models used do not represent the clinical course in human sepsis, which has to be regarded as a disadvantage of these models. TNF-α, on the one hand, is well known to activate NF-κB, which in turn leads to a strong induction of cyclooxygenase-2 (COX-2) expression. Prostaglandins on their part (progressively more produced after COX-2 induction) have been shown to stimulate glycogenolysis in the liver [43]. On the other hand, TNF-α and IL-1β have been demonstrated to induce hypoglycemia [44]. Therefore, as LPS treatment, followed by PCI and CLP, had the largest impact on glucose levels, TNF-α kinetics may serve as an explanation for the differences observed in the glucose values over time between the three animal models. Additionally, the acute inflammatory process caused by LPS administration may have led to rapid glucose consumption, further contributing to the rapid decline in blood glucose values in this model.

In septic patients, the liver biotransformation capacity is of essential importance for the detoxification and elimination of toxic endogenous metabolites as well as exogenously administered antibiotic therapies. Lower CYP activity leads to a reduction in metabolized compounds that can be then excreted via bile and the kidneys, leading to an accumulation of these compounds and toxic effects, exerting a negative impact on the already reduced health condition of the patients [45]. TNF-α and IL-6 play a pivotal role in impairment of liver function by decreasing the biotransformation capacity [46, 47]. Both LPS and PCI treatments induced early increases in TNF-α and IL-6 serum levels, followed by a maximal loss in CYP enzyme activity and expression at 12–24 h. Also here, after 72 h baseline values were reached again. In contrast, CLP-treated mice exhibited decreased CYP activity and expression at later time points, following the prolonged release of TNF-α and IL-6 in this model. As CLP treatment decreased CYP activities at later time points, the prolonged impairment of health status in these animals may be, at least in part, explained by the reduced ability to detoxify endogenous substances, generated as a result of the inflammatory condition.

Pro-inflammatory cytokine expression leads to increased generation of reactive oxygen species (ROS) via impairment of mitochondrial integrity and function and increased nicotinamide adenine dinucleotide phosphate (NADPH)-oxidase expression and activity [48, 49]. Elevated serum TNF-α, IL-6, and IFN-γ levels could have led to the decreased GSH/GSSG and increased LPO values seen in all models and in most organs investigated in this study. These parameters are indirect indicators of increased oxidative stress due to elevated production of ROS. While the glutathione system serves as an important anti-oxidative means of the body with, however, a limited capacity, LPO are the result of an excessive oxidative stress which had overwhelmed the overall antioxidant capacity of the organism and had caused (besides a damage of other macromolecules) a degradation of cellular membranes. The severity of oxidative stress may have been additionally augmented by the diminished total glutathione content of both the liver and the other organs investigated (Additional File 2), indicating a reduced capacity to produce glutathione and export this important antioxidant to other tissue sites. This may have been caused either by cytokine-mediated downregulation of glutathione synthesizing/recycling enzymes or by direct damage of liver cells, as indicated by elevated serum ALAT values in all three animal models.

Whereas the GSH/GSSG ratio and total glutathione content in LPS- and PCI-treated organs returned to baseline at later time points, in the CLP model, values progressively decreased with the course of treatment in all organs investigated. These observations were paralleled by serum ALAT levels. The activities remained increased at 48 and 72 h after CLP treatment, whereas LPS- and PCI treated-mice displayed normal ALAT values at the end of the experimental time course. TNF-α, IFN-γ, and IL-6 serum levels (and therefore probably ROS levels) were increased at later time points after CLP, which differed from LPS and PCI treatment. ROS have been shown to negatively impact biotransformation capacity [50]. Accordingly, in contrast to the LPS and PCI models, liver CYP expression and activity in CLP-treated mice also remained lower at later time points, as mentioned above.

Perhaps the increased activity and expression of HO-1 after LPS and PCI treatment has contributed to the observed recovery in these models. Targeted overexpression of HO-1 has been demonstrated to have beneficial effects in various experimental animal models of inflammation [51]. Moreover, previous investigations have shown that HO-1 attenuates TNF-α and ROS levels [52], which agrees with our results after LPS and PCI treatment. Additionally, HO-1-deficient mice have been shown to be more susceptible to polymicrobial sepsis, underlining the protective role of this enzyme [51, 53]. As CLP treatment did not affect the expression and activity of this anti-oxidative and anti-inflammatory enzyme, this might have been a contributing factor to the protracted and delayed disease seen in this model. Moreover, the sustained increase in ALAT levels underlines the limited liver function at later time points in the CLP model. Increased GSH and HO-1 levels might have contributed to the recovery seen in the LPS- and PCI-treated mice by triggering compensatory mechanisms [54].

As TNF-α and IFN-γ have been previously shown to be able to strongly upregulate inducible nitric oxide synthase (iNOS) [55], we stained iNOS in the livers and spleens. We found an evident amount of positive neutrophils having immigrated into the liver and splenic tissue after LPS treatment, which has also been reported by others [17]. This infiltration, although to a lesser extent, was also observed after PCI treatment, whereas the CLP procedure resulted in only few neutrophils in the liver tissue. Previous studies have shown that pro-inflammatory cytokines enhance the expression of cell adhesion molecules after bacterial infection [56]. Accordingly, more neutrophils could have entered the tissue due to an increased amount of adhesion molecules in the vessels. Lower amounts of pro-inflammatory cytokines were expressed in the PCI treatment group compared to the LPS group, and even less after CLP treatment. Therefore, fewer cells may have migrated as a result of lower cytokine levels in these models.

Previous studies have shown that high doses of LPS may trigger cell death pathways [57]. However, we did not see a significant expression of cleaved caspase-3 in the liver, regardless of treatment. In our study lower doses of LPS and fecal slurry were administered, which may not have been high enough to illicit an apoptotic response in the liver. Nevertheless, the livers of LPS-treated mice showed remarkable amounts of fat accumulation and periportal inflammatory cell infiltration, suggesting the massive TLR4 activation to be relevant for these effects. In contrast to the liver, however, increased cleaved caspase-3 expression was detectable in the spleen. Again, the increased production of TNF-α, IL-6, and IFN-γ, as well as high mobility group box-1 (HMGB-1) might have been responsible for this effect [35, 58]. Interestingly, CLP-treated mice displayed the highest cleaved caspase-3 expression early after treatment. This may be a result of an endotoxin and TNF-α-independent pathway, as has been proposed previously [59].

Interestingly, splenomegaly occurred after LPS and PCI treatment, but CLP had no impact on spleen mass. This is in agreement with a previous study that revealed septic spleens to exhibit increased weights 2–4 weeks after CLP onset [35]. The massive splenic erythrocyte accumulation in endotoxin and PCI-treated mice more than likely contributed to spleen enlargement. This is further supported by our previous report that detected a decreased hematocrit in LPS-treated mice after 24 h [19]. LPS and PCI challenge has led to a redistribution of erythrocytes into the spleen, which may indicate damage to the red blood cells. Infiltrating immune cells, predominantly monocytes and neutrophils, could also have contributed to increased splenic weights. CXCR4, CXCL12, TNF-α, iNOS, CD68, and CD8+ cells were detected in the red pulp of LPS and PCI-treated mice after 72 h. Based on histological appearance, we believe that these cells were predominantly monocytes and neutrophils, which had entered the tissue, perhaps to start the recovery process following the onset of inflammation.

As chemokines are of great importance for migration, and as we observed immune cell infiltration into the tissues, the chemokine receptor CXCR4 expression was examined. Here, our previous observations were confirmed, that CXCR4+ cells undergo apoptosis in the white pulp and are engulfed by macrophages, which then appear as tingible body macrophages [33]. Especially LPS- and PCI-treated mice exhibited a large amount of CXCR4+ cells in the red pulp after 72 h. Other authors have shown that LPS induces a reduction in CXCR4 surface expression in a dose- and time-dependent manner in peripheral neutrophils and monocytes [60]. In the current study, these findings can be explained by increased emigration of these cells out of the circulation and into the tissues. Consistent with this, CXCL12 levels were decreased in the serum, suggesting lower retention of CXCR4+ cells in the blood and an increase in CXCR4+ cells in the tissues. Additionally, in the literature treatment with TNF-α, IFN-γ, IL-1β and LPS has been shown to downregulate CXCL12 and CXCR4 expression in human brain microvessel endothelial cells [61], suggesting TLR4 activation (along with the subsequent pro-inflammatory cytokine release) to be the major activator. This might be a reason why we have observed noticeable effects after LPS or PCI treatment only.

To evaluate the anti-inflammatory response, we measured the serum levels of IL-10, as a prototypic anti-inflammatory cytokine. Consistent with our other results and with findings in the literature [62], CLP treatment induces anti-inflammatory mechanisms late in the course of disease. The early IL-10 induction after LPS and PCI treatment may have contributed to the early recovery observed in these two animal models, as IL-10 has been shown to suppress the production of TNF-α, IL-1, IL-6, and IL-8 [63, 64]. Presumably, the acute inflammatory stimulus in LPS or PCI treated mice has triggered the early IL-10 release.

## Conclusions

In summary, the present study demonstrates that non-lethal, systemic inflammation induced by LPS and PCI treatment influences organ functions, cytokine responses, oxidative stress levels, and overall health status differently in comparison to the CLP procedure. The LPS and PCI models induced rapid onset of inflammation, including an early increase in serum pro-inflammatory cytokines and oxidative stress in organ tissues, a rapid decrease in blood glucose values and biotransformation capacity, immune cell infiltration from the circulation into the liver and spleen, and apoptosis in the spleen. LPS administration exhibited the strongest inflammatory effects of all of the models tested. Interestingly, LPS- and PCI-treated mice recovered by 72 h, probably through the induction of protective mechanisms, such as HO-1 upregulation. CLP treatment induced a protracted course of disease with impairment of health status persisting through the end of the experiment.

This study demonstrates that it is essential to be aware of differences in models of systemic inflammation to ensure that experimental aims are addressed appropriately. This includes an evaluation of the time period to be investigated when testing new anti-inflammatory drugs, for example. In assessing the impact on acute inflammation, the LPS model is the most suitable, as systemic effects are easily identifiable and measureable. In addition, LPS is easy to administer and the model is highly reproducible. Certainly, injection of LPS does not exactly reproduce the course of human sepsis, while CLP serves as the “gold standard” in this respect. However, with this model the time period has to be chosen long enough as the treatment induces a delayed course of inflammation. In conclusion, each model has benefits which must be weighed with the experimental parameters to be investigated and the overall aim of each individual study.

## Additional files

Additional file 1: Blood pressure, heart rate, body weight and body temperature. At the time point indicated, mice were sacrificed and body weights as well as body temperatures were determined. Additionally, blood pressure values and heart rates were assessed after 24 h. Data are given as mean ± standard deviation (SD); *n* = 4–6 for each group and time point. Statistical significance was determined by using the non-parametric Kruskal-Wallis test, followed by pairwise Mann-Whitney U tests. Statistical comparisons were made versus the control of each group and are denoted as follows: LPS (asterisk, *), PCI (plus, +), CLP (diamond, #). A *p* value <0.05 (*,+,#) was considered statistically significant; a *p* value <0.01 (**,++,##) and a *p* value <0.001 (***,+++,###) are further specified. (TIFF 422 kb)

Additional file 2: Total glutathione concentration in different organs, glutathione-S-transferase activities and F4/80 expression in the livers. At the time point indicated, mice were sacrificed and different organs were collected for the analysis of the total glutathione content (**a-d**). Furthermore, glutathione-S-transferase (GST) activities were determined in the 9000 g liver supernatants (**e**). Data are given as mean ± standard deviation (SD); *n* = 4–6 for each group and time point. Statistical significance was determined by using the non-parametric Kruskal-Wallis test, followed by pairwise Mann-Whitney U tests. Statistical comparisons were made versus the control of each group and are denoted as follows: LPS (asterisk, *), PCI (plus, +), CLP (diamond, #). A *p* value <0.05 (*,+,#) was considered statistically significant; a *p* value <0.01 (**,++,##) and a *p* value <0.001 (***,+++,###) are further specified. The photomicrographs in (**f**) show representative livers after 48 h, displaying large amounts F4/80 positive cells after LPS and PCI treatment. In (**g**), HE stainings of PCI- and CLP-treated mice after 24 h are shown as a supplement to Fig. 3d. PCI and CLP treatment caused almost no fat accumulation in the livers. (TIFF 1084 kb)

Additional file 3: iNOS expression in the livers and spleens as well as HE staining in the spleens of PCI-treated mice. At the time point indicated, mice were sacrificed and livers and spleens were collected for immunohistochemical analysis. (**a**) Course of iNOS expression (red-brown color, counterstaining with hematoxylin; original magnification: 400×) in the livers of PCI-treated mice as supplements to Fig. 4a and b. Arrows exemplarily show the infiltrating neutrophils. (**b**) HE-stained spleens 24 h after PCI treatment (original magnification: 400×). (**c**) iNOS expression patterns after PCI treatment at 24 h (original magnification: 400×) as a supplemental to Fig. 6e. (TIFF 1827 kb)

## Abbreviations

ALAT: Alanine aminotransferase
CD: Cluster of differentiation
CLP: Cecal ligation and puncture
CSS: Clinical severity score
CXCL12: CXC motif chemokine 12
CXCR4: CXC chemokine receptor type 4
CYP: Cytochrome P450
ECOD: Ethoxycoumarin-O-deethylation
EMND: Ethylmorphine-N-demethylation
EROD: Ethoxyresorufin-O-deethylation
GSH: Reduced glutathione
GSSG: Oxidized glutathione
GST: Glutathione-S-transferase
HO-1: Heme oxygenase 1
IL: Interleukin
iNOS: Inducible nitric oxide synthase
LPO: Lipid peroxides
LPS: Lipopolysaccharides
MROD: Methoxyresorufin-O-demethylation
PCI: Peritoneal contamination and infection
PROD: Pentoxyresorufin-O-depentylation
SIRS: Systemic inflammatory response syndrome
TBARS: Thiobarbituric acid-reactive substances
TLR4: Toll-like receptor 4
TNF: Tumor necrosis factor
