# Supplementary figures and images for: Comprehensive comparison of three different animal models for systemic inflammation
Source: J Biomed Sci. 2017 Aug 24;24:60. doi: 10.1186/s12929-017-0370-8 (PMC5569462; doi:10.1186/s12929-017-0370-8)

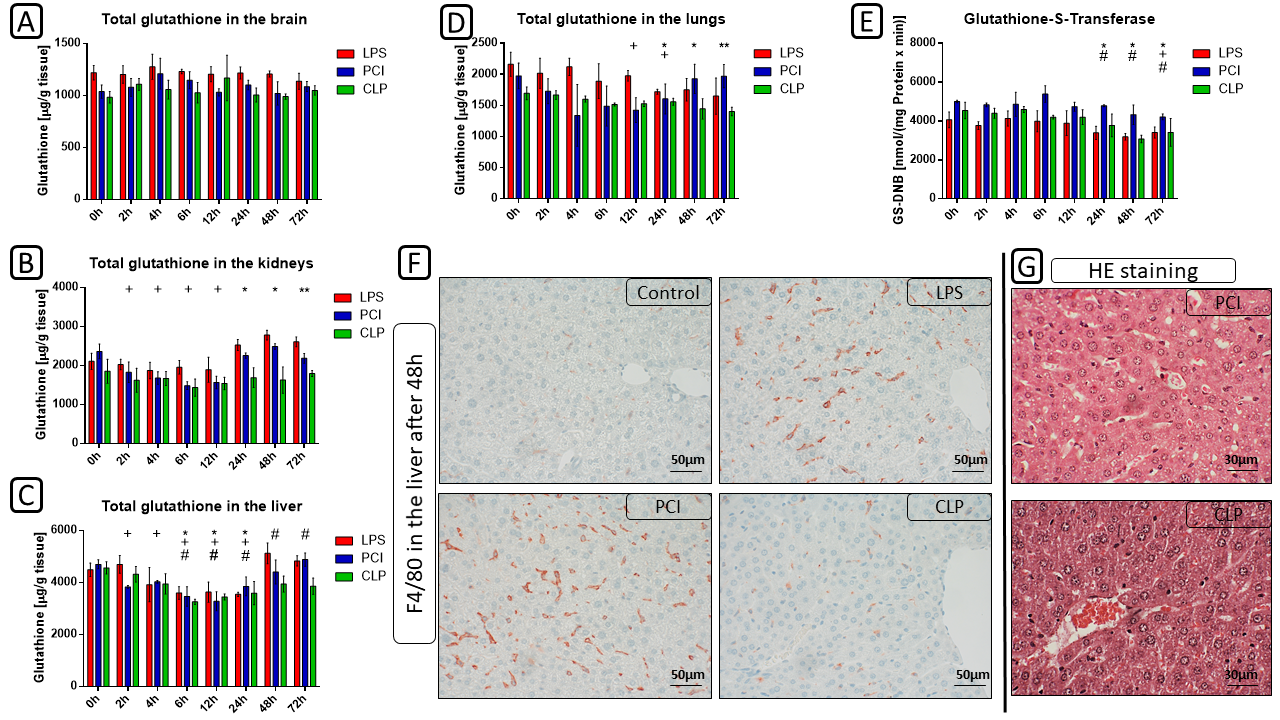

Supplement: Supplementary file 2 — Total glutathione concentration in different organs, glutathione-S-transferase activities and F4/80 expression in the livers. At the time point indicated, mice were sacrificed and different organs were collected for the analysis of the total glutathione content (a-d). Furthermore, glutathione-S-transferase (GST) activities were determined in the 9000 g liver supernatants (e). Data are given as mean ± standard deviation (SD); n = 4–6 for each group and time point. Statistical significance was determined by using the non-parametric Kruskal-Wallis test, followed by pairwise Mann-Whitney U tests. Statistical comparisons were made versus the control of each group and are denoted as follows: LPS (asterisk, *), PCI (plus, +), CLP (diamond, #). A p value <0.05 (*,+,#) was considered statistically significant; a p value <0.01 (**,++,##) and a p value <0.001 (***,+++,###) are further specified. The photomicrographs in (f) show representative livers after 48 h, displaying large amounts F4/80 positive cells after LPS and PCI treatment. In (g), HE stainings of PCI- and CLP-treated mice after 24 h are shown as a supplement to Fig. 3d. PCI and CLP treatment caused almost no fat accumulation in the livers. (TIFF 1084 kb) [file 12929_2017_370_MOESM2_ESM.tif]

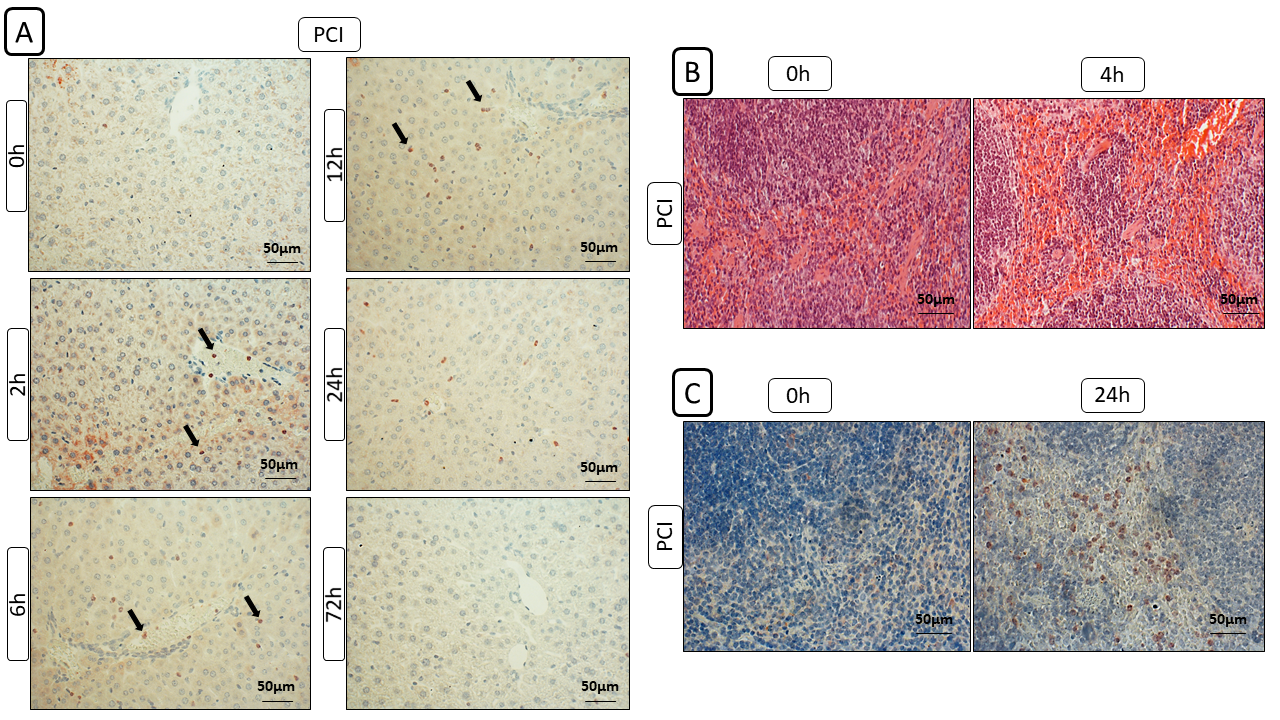

Supplement: Supplementary file 3 — iNOS expression in the livers and spleens as well as HE staining in the spleens of PCI-treated mice. At the time point indicated, mice were sacrificed and livers and spleens were collected for immunohistochemical analysis. (a) Course of iNOS expression (red-brown color, counterstaining with hematoxylin; original magnification: 400×) in the livers of PCI-treated mice as supplements to Fig. 4a and b. Arrows exemplarily show the infiltrating neutrophils. (b) HE-stained spleens 24 h after PCI treatment (original magnification: 400×). (c) iNOS expression patterns after PCI treatment at 24 h (original magnification: 400×) as a supplemental to Fig. 6e. (TIFF 1827 kb) [file 12929_2017_370_MOESM3_ESM.tif]
